# 741 Implementation and Effectiveness Outcomes of Geriatric Burn Bundle at a Regional Burn Center

**DOI:** 10.1093/jbcr/irad045.216

**Published:** 2023-05-15

**Authors:** Tam Pham, Carolyn Blayney, Kathleen O'Connell, Julia Oehlers, Barclay Stewart, Saman Arbabi, D Andrew Tucker

**Affiliations:** University of Washington, Seattle, Washington; Harborview Medical Center, Sammamish, Washington; University of Washington, Seattle, Washington; University of Hawaii, Honolulu, Hawaii; University of Washington, Seattle, Washington; University of Washington and Harborview Medical Center, Seattle, Washington; Swedish Medical Center, Seattle, Washington; University of Washington, Seattle, Washington; Harborview Medical Center, Sammamish, Washington; University of Washington, Seattle, Washington; University of Hawaii, Honolulu, Hawaii; University of Washington, Seattle, Washington; University of Washington and Harborview Medical Center, Seattle, Washington; Swedish Medical Center, Seattle, Washington; University of Washington, Seattle, Washington; Harborview Medical Center, Sammamish, Washington; University of Washington, Seattle, Washington; University of Hawaii, Honolulu, Hawaii; University of Washington, Seattle, Washington; University of Washington and Harborview Medical Center, Seattle, Washington; Swedish Medical Center, Seattle, Washington; University of Washington, Seattle, Washington; Harborview Medical Center, Sammamish, Washington; University of Washington, Seattle, Washington; University of Hawaii, Honolulu, Hawaii; University of Washington, Seattle, Washington; University of Washington and Harborview Medical Center, Seattle, Washington; Swedish Medical Center, Seattle, Washington; University of Washington, Seattle, Washington; Harborview Medical Center, Sammamish, Washington; University of Washington, Seattle, Washington; University of Hawaii, Honolulu, Hawaii; University of Washington, Seattle, Washington; University of Washington and Harborview Medical Center, Seattle, Washington; Swedish Medical Center, Seattle, Washington; University of Washington, Seattle, Washington; Harborview Medical Center, Sammamish, Washington; University of Washington, Seattle, Washington; University of Hawaii, Honolulu, Hawaii; University of Washington, Seattle, Washington; University of Washington and Harborview Medical Center, Seattle, Washington; Swedish Medical Center, Seattle, Washington; University of Washington, Seattle, Washington; Harborview Medical Center, Sammamish, Washington; University of Washington, Seattle, Washington; University of Hawaii, Honolulu, Hawaii; University of Washington, Seattle, Washington; University of Washington and Harborview Medical Center, Seattle, Washington; Swedish Medical Center, Seattle, Washington

## Abstract

**Introduction:**

Strong evidence supports geriatric consultation and targeted management in the care of older injured patients. We developed and implemented a geriatric care bundle that includes clinical frailty screening, mini-nutritional assessment and feeding protocol, and reduction in initial prescribed opiate doses for patients ≥65 years. Clinical frailty scores and co-morbidities trigger consultation to geriatric and palliative care services rather than chronological age. We sought to evaluate process adherence, length of stay (LOS) and mortality as measures of bundle implementation and effectiveness.

**Methods:**

We performed a retrospective medical record review of older burn-injured patients aged ≥65 years hospitalized at a regional burn center over the first two years of implementation (July 2019 to June 2021). We reviewed patient and injury characteristics. Primary outcome was adherence to specific bundle components: clinical frailty scale (CFS-9) and mini-nutritional screening rates, and geriatric and palliative consultation rates, stratified by implementation year. Secondary outcomes included LOS and mortality.

**Results:**

All 127 older adult patients admitted to the acute burn service were analyzed. Median age was 71 years (IQR67-78), median burn size was 6% TBSA (IQR2-13), median frailty score was 3 (IQR3-4), and median mini nutrition score was 11 (IQR9-14). CFS-9 scoring and mini nutrition completion rates, as well as geriatric service consultations increased over 2 years (Table). In contrast, palliative care consults decreased over 2 years (from 34% to 16%). LOS and mortality decreased each year from 9 to 7 days and 14 to 10%, respectively.

**Conclusions:**

Implementation of a geriatric burn bundle demonstrated a sustained increase in utilization of geriatric focused interventions, tailored to patients most likely to benefit based on frailty and comorbidities. Electronic medical records system integration during the initial implementation phase of the geriatric burn bundle was effective in obtaining high compliance and completion rates. However, there is still progress to be made toward full compliance with screening.

**Applicability of Research to Practice:**

Implementation of a targeted bundle should be considered for optimal geriatric burn care.

